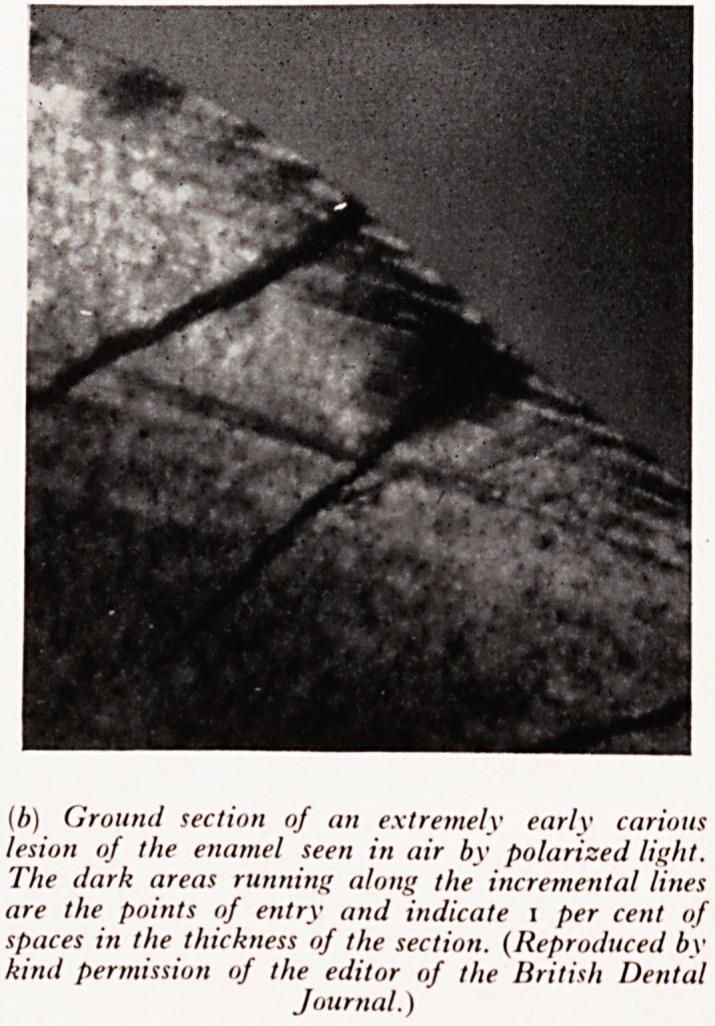# Decay of the Teeth
*Long Fox Memorial Lecture, delivered on 28th October, 1958.


**Published:** 1959-07

**Authors:** A. I. Darling

**Affiliations:** Professor of Dental Medicine, University of Bristol


### DECAY OF THE TEETH*

BY

A. I. DARLING, D.D.SC., M.D.S., F.D.S.R.C.S., L.R.C.P., M.R.C.S.

*Professor of Dental Medicine, University of Bristol*

Dental decay, or dental caries as it is more properly called, is a disease which, as everyone knows, produces holes or cavities in the teeth. These occur where the tooth surfaces are protected from the normal cleansing action of the tongue, the lips and the cheeks or where the softer tissues of the root (Plate XXII) are exposed by recession of the gums with increasing age. There are three main sites: in the natural folds found in the biting surfaces of the posterior teeth, at the points of contact between the teeth, and at the junctions between the teeth and the gums. For convenience these are called occlusal, interproximal, and cervical danger areas (Fig. 1).

FIG. 1. *The "Danger Areas" for dental caries. These are stagnation areas around the teeth and include interdental and cervical areas and occlusal fissures.*

### *Stages of Decay*

The lesion itself is first recognized, if it can be seen at all, as a small whitish patch in the normally translucent enamel. This does not mean that all whitish patches in the enamel are carious, but rather that all whitish patches occurring in the danger areas are very probably carious. The white patch gradually increases in size and the normally hard and glossy enamel surface becomes roughened. At the same time the lesion may become stained. Soon after the roughness is noticed the enamel surface in the centre of the lesion may break down to form a small cavity. This then progresses in size until the whole crown of the tooth may be involved(Plate XXIII(*a*)).

Because two of the three usual stagnation areas are in well protected sites, it is

*Long Fox Memorial Lecture, delivered on 28th October, 1958.

very difficult to find the early lesions and they cannot be recognized clinically until cavities begin to form. Fortunately radiographs help us to find some of these lesions at a fairly early stage, but we are now beginning to understand that any lesion which can be found by probe or radiography has already done much damage (Plate XXIII(*b*)).

*Symptoms and Complications*

At the same time as this process is occurring the patient begins to notice certain symptoms. About the time that the early cavity begins to form, the tooth may become sensitive to sweet foods and possibly also to salty foods. Such pain is at first transient, disappearing with the removal of the stimulus, but as the cavity increases in size and the disease penetrates further into the tooth the soft tissue in the centre of the tooth, which we call the pulp or nerve, becomes inflamed. This is slight at first but it results in an increased sensitivity of the tooth to sweet foods and painful sensation from heat or cold. As the condition progresses the pain becomes more intense and lasts longer until eventually the inflammation of the pulp becomes so severe that the pain is continuous. It mounts in intensity because of the increasing pressure within the pulp until the patient is at last driven to seek relief in one way or another. Fortunately, this final stage of acute pulpitis rarely lasts more than half to one hour, as the pressure within the pulp causes an obstruction of the blood flow and the pulp tissue dies. This is usually noticed as a sudden cessation of pain and often occurs as the patient rings the dentist's door bell.

At this stage of acute pulpitis the tooth is usually past saving for almost inevitably the inflammation will have passed beyond the tooth into the supporting structures (Fig. 2). Once this has happened, sooner or later it leads to the formation of a dental

FIG. 2. *The arrangement of the teeth and supporting structures.*

abscess around the apex of the root. This in turn may cause considerable pain extended over several months if it becomes chronic. Finally it discharges and the pain is relieved. The infection however persists and a discharging sinus usually remains connecting the root of the carious tooth to the surface of the gum or face. In this stage the patient suffers no real pain except in acute exacerbations of the abscess.

The whole process from the onset of caries may take several years or may occur much more rapidly. Throughout this time the patient suffers periodic attacks of pain often being subject to some pain almost continuously. As for the infection, it is certain that this can at times cause systematic disease, but it seems unlikely that it causes much more than malaise and misery in most patients.

Fortunately today many of the sequelae of dental caries are prevented by good dental treatment at an early stage of the disease, but there are still far too many patients who prefer to avoid conservative treatment and wait till their teeth "fall out" or require extraction.

Having examined the natural history and complications of the disease we must now consider its scope.

*Incidence*

For many years now the percentage of school children in this country who are clinically free from caries has been 5 per cent or less. Being based only on clinical examination this simply means that they have no recognizable cavities and the figure is therefore probably too high. It is also known that in adults there are even fewer who have never had caries. This pattern is typical of most European people and their off-shoots in North America and Australia, while in New Zealand the condition of the teeth is even worse. Reliable figures are difficult to obtain from Asia but there seems little doubt that the caries incidence among Russians, Chinese and Indians is low, while in Africans living on their natural diet and in Eskimos before the arrival of trading posts, caries was almost non-existent.

Historically the advance of caries can be closely related to civilization. It is very rare in wild animals and in primitive man, but with the advent of each civilization its incidence seems to have increased. It was certainly known to the Egyptians, though probably it was not very widespread. By the time of the Romans it was already a matter of some importance and dental restorations were being used. In Britain during the Middle Ages it was quite commonplace. Since then it has steadily advanced until the present day.

Today we accept the fact that the schoolchild usually has at least one cavity for each year of life. On average each schoolchild produces one or two cavities each year and this may begin as early as 2 to 3 years of age or within one year of cutting the first tooth. This however is extreme, but it must not be forgotten that this picture is probably too optimistic for we can only count cavities. The early lesions are often inaccessible and therefore unrecognized. There are several cases on record where it has been necessary to extract all teeth for children of three to four years, solely because of caries. The teeth themselves showed no gross faults of structure. In fact the common story of chalky teeth is usually only an unsatisfactory excuse.

It is often suggested that pregnancy and other systemic conditions may increase the incidence of caries. There is no doubt that the structure of developing teeth may be affected by systemic disease, rendering them more susceptible to caries, but there is no evidence that the structure of the enamel of fully developed and erupted teeth can be modified. As for the relationship between pregnancy and caries, the evidence shows quite clearly that the incidence of caries in women of child bearing age shows no significant difference whether they have many, few or no children. It is therefore unreasonable to regard pregnancy as a cause of caries, though it is still wise for expectant mothers to have good dental treatment because of the possibility of pregnancy gingivitis and the desirability of avoiding radical dental treatment in late pregnancy.

An examination of the graph of caries incidence in this country shows that having started early, the number of new cavities steadily increases each year till a peak is reached roughly between the ages of 9 and 14 years. It then falls, probably because most of the danger zones are already carious and reaches a steady low incidence in the late 'teens. This is maintained until middle life when as the gums recede new cavities begin to occur in the exposed roots. This cervical caries of middle age is relatively a minor problem. The major problem is the loss of teeth in childhood, for it is now known that the premature loss of baby teeth and of permanent teeth in early life can cause many dental deformities and can contribute to the causation of pyorrhoea in later life.

It has now become a matter of national importance that caries should be prevented. Whether we consider it as a financial matter of the cost of treatment under the health service or as the loss of man hours from toothache and treatment, or simply as a question of pain and misery, it still affects everyone and causes altogether too much trouble.

If it is to be prevented then we must know something of its causes and pathology.

*Pathology*

The first real attempt to understand the process was made by Miller in 1890. He suspected that caries was caused by the fermentation of food stagnating around the teeth. To demonstrate this he took pieces of teeth and incubated them in a mixture of bread and saliva and eventually produced the first artificial caries, with lesions in many ways similar to the natural variety. On these experiments he built the theory that caries is caused by acid which is produced by the action of bacteria on refined carbohydrates stagnating round the teeth. This theory is still acceptable today with a few modifications.

Interest has always centred on the attack of caries upon the enamel for this is the tissue which must first be breached before the tooth is endangered. Unfortunately, in Miller's day it was thought that enamel consisted wholly of calcium salts, but about 1906 Bodecker demonstrated that it also contained a small amount of organic or protein material.

It had been recognized for some time that Miller's theory did not explain all the facts and when it was discovered that the protein or organic matrix showed distinct changes in caries a new theory was advanced. Caries was now said to be caused by bacterial attack upon the protein.

Further experimentation has now shown quite clearly that two processes are involved. These are the removal of calcium salts and the alteration and destruction of the organic matrix. It is also clear that caries cannot occur without the stagnation of refined carbohydrates and without the presence of bacteria. It is highly probable that acid is produced which removes the calcium salts, but other methods are at present being suggested.

*Prevention*

On this basis several methods of prevention have been tried. In animals susceptible to caries it has been shown conclusively that if bacteria can be excluded from the mouth no caries occurs, but in man it has been shown equally conclusively that while antiseptics and antibiotics will eliminate the organisms almost completely for a short time after their application the organisms return within the hour as the drugs are washed away by the saliva. The same is true of various chemicals which have been used to neutralize the acid and in addition there is considerable difficulty in getting the neutralizing agent to the sites where the acid is produced.

It is also known that where patients have been willing to exclude refined carbohydrates completely from their diets the caries incidence falls very rapidly and it seems quite clear from the evidence that most of the general variations in caries incidence with race or civilization can be explained by the amount of refined carbohydrate in the diet.

Many dental surgeons have limited the intake of refined carbohydrate by their children with a startling lack of caries as the result. Unfortunately even these children grow out of parental control and may become confirmed suckers of sweets between meals. Then unfortunately the caries begins. This is the real problem. How can dental

PLATE XXII

*Ground section of tooth showing the body of the tooth consisting of dentine. The crown is covered by a layer of enamel which shows diagonal incremental lines. The pulp cavity is cut rather obliquely and should continue right through the apex of the root.*

(*a*) *The clinical stages of caries in interproximal and occlusal surfaces. The early stages are almost invisible.*

(*b*) *Dental radiographs showing caries of the interproximal surfaces at various stages in a child.*

(*a*) *Soft radiograph of a ground section of carious enamel. The surface zone is apparently unaffected though there is marked decalcification beneath it.*

(*b*) *Ground section of early caries of the dental enamel. This shows the affected enamel surrounded by a characteristic dark zone. The translucent zone is hardly visible but usually lies between the dark zone and the normal enamel.* (*Reproduced by kind permission of the editor of the British Dental Journal.*)

(*a*) *Soft radiograph of carious enamel. This lesion is well established and now shows the points of entry through the surface zone, related to the incremental lines.* (*Reproduced by kind permission of the editor of the British Dental Journal.*)

(*b*) *Ground section of an extremely early carious lesion of the enamel seen in air by polarized light. The dark areas running along the incremental lines are the points of entry and indicate* 1 *per cent of spaces in the thickness of the section.* (*Reproduced by kind permission of the editor of the British Dental Journal.*)

caries be prevented without a serious change in our habits, for it would seem that the public do not yet regard dental disease as of sufficient importance to justify a change of habits.

Much can be done by the individual to reduce the stagnation of food around the teeth by tooth brushing and mouth rinsing. The latter is very valuable and consists of squirting water back and forward between the teeth immediately after meals. No-one would claim that this does more than reduce the incidence of caries and even so children present the real problem as it is difficult to get their co-operation in these exercises without the immediate supervision of an adult or until they become susceptible to reason, which is often after much damage has been done.

*Fluorides*

Perhaps the most important of all preventive measures has been the use of fluorides. Before the war it was noted that where the population had mottled teeth the caries incidence in children was only half that of similar communities without mottling. It was later discovered that the mottling was caused by a high concentration of fluorides in the water supply. Further investigation showed that the fluorides were also responsible for the reduction in caries incidence and that the same effect could be obtained without mottling by using one part per million of fluoride in the drinking water.

Many communities in America have been adding this amount of fluorides to their drinking water for periods of 10 years and more and they have consistently reduced the incidence of caries in their children by about 50 per cent. Recently a similar procedure has been tried in 5 centres in this country but it is too early to expect results. It is however confidently expected that similar results will be obtained. Unfortunately there has been a great deal of acrimonious and uninformed discussion of the dangers and even of the ethics of this procedure, but we are not concerned with these aspects in this paper.

Our interest lies in two facts. We are told that fluorine renders the calcium salts of the enamel much less soluble but only produces a 50 per cent reduction. We are also discovering that one of the principal features of the mottled teeth of gross fluorosis, which show little caries, is that they are badly calcified.

If our previous theories are correct poorly calcified teeth should be more susceptible to caries and not less. Nor are these the only badly calcified teeth to be unusually free from caries. We have recently had the opportunity of examining a series of 25 cases with a rare disease of the enamel called amelogenesis imperfecta, which is often hereditary and unrelated to fluorine. These teeth are also badly calcified and yet the incidence of caries in these patients was also remarkably low.

It would seem that we know little of the mechanism by which fluorides reduce the incidence of caries and indeed we know little of the process of caries.

*Progress in Research*

Too often in dentistry the process of disease has been guessed at from the appearances seen down a microscope, instead of finding new techniques which will positively identify the process. Too frequently sound observations have been ignored so that a convenient theory could be sustained.

Our work on caries began from two facts and two problems. It is well known to all dentists that caries develops at the contact points between teeth and that of the two surfaces in contact one may become carious while the other does not. At times the surface which becomes carious has been exposed to the cariogenic agents for less time than the non-carious surface. If the theories are correct both should become carious as they are subject to the same environment. The fact that they did not suggests that the structure of the tooth may be of importance.

The theory that acid formed from the fermentation of carbohydrates attacks the tooth, suggests that there should be a gradual erosion of the enamel, but as long ago as 1932 it was shown that when sections of early carious lesions were ground and X-rayed to see where the decalcification had occurred, a thin surface zone of enamel remained almost unaltered while the enamel beneath was decalcified (Plate XXIV(*a*)). This again implied a modification of the process by the structure of the enamel.

It had also been known for many years that two processes were involved in caries. The removal of calcium salts and the breakdown of the organic matrix. But while each school of thought had its own idea of which happened first, no-one seemed to know.

The appearances of the early carious lesions when seen down a microscope in ground sections of teeth, showed two zones surrounding the main lesion (Plate XXIV(*b*)). A dark zone next to the lesion which no-one could explain, and a translucent zone outside it which had often been described as a laying down of additional calcium salts by the tooth to resist the progress of the disease.

The investigations cannot be described at length in this paper, but some of the results are of interest.

Since the beginning of dental histology, the usual medium for mounting ground sections of teeth has been Canada Balsam. The section which is ground in water must be washed in alcohol and Xylol before mounting in the balsam. It was found that by using a watery medium not requiring the washing in alcohol the dark zone and the translucent zones disappeared. Further investigations with polarized light showed that these zones were caused by small spaces some of which became filled with balsam to become translucent and some probably with bubbles of gas to make them refract differently from the normal enamel giving the dark opaque appearance. This finding was at first accidental, but it is surprising that it had never been reported before, for watery media have been used in biology for many years.

The next step was to try to establish the nature of the early carious lesion and to decide whether the removal of the calcium salts occurred before the recognizable changes in the protein of the organic content or whether, as some thought, the reverse was true.

To do this we examined the evidence and discovered that most of the lesions in which organic change had been demonstrated were quite advanced and cavities were present. It was also apparent that some other changes were present which were well in advance of the changes in the organic material. It seemed highly probable that these earlier changes were caused by the removal of calcium salts.

Some very early lesions were then taken and split through the centre. From one half, ground sections were prepared and microradiographed for evidence of decalcification. The other halves were prepared and stained to show changes in the organic part of the enamel. In this way it was shown that calcium salts had been removed from these lesions before any change in the organic or protein part could be seen. Further investigation on these lines is still going on and we now know that decalcification occurs very early in caries. It is then followed by organic change at about the time when the surface begins to break down and soon after this the bacteria start to enter the enamel structure. Until this stage the bacteria have been producing their effects from a distance.

The greatest problems still remained. We could not yet explain how the carious process passed through the enamel surface zone without apparently affecting it and yet was able to cause a considerable loss of calcium salts within the deeper enamel. We had confirmed the previous evidence of a resistant surface layer and came to the conclusion that the process must enter through minute faults or openings which so far had not been demonstrated. It was obvious that the next step was to find these openings. We formed the opinion that though the openings must be extremely small at first they would probably increase as the lesion progressed. As a result we began looking at the more advanced lesions just before cavities would be expected. As is so often the case they were found when we had almost given up hope. They showed on fine X-rays of the ground sections as small openings related to the developmental pattern of the enamel and consisted of several small openings rather than the single point of entry we had envisaged (Plate XXV(*a*)). At this stage we estimated that about 5 per cent of the calcium salts had been removed in these openings, while 25 per cent to 50 per cent had been removed in the underlying lesion. We were anxious to demonstrate them at an earlier stage and eventually, with the aid of biologists and physicists, devised a means for demonstrating the spaces from which material had been removed; first at about 5 per cent and later as low as 1 per cent of spaces. (Plate XXV(*b*)). The lesions with one per cent of spaces could not be found in even the most careful macroscopic examination, so they had to be sought in apparently sound teeth. Eventually such lesions were found and they showed the points of entry in precisely the same parts of the developmental pattern as they occupied in the later lesions seen by fine X-rays. At this early stage we could only recognise the spaces. We could not be sure what had been removed from them. It was therefore necessary to add an earlier stage to the process of caries. This stage occurs before recognizable decalcification, and is the production of minute spaces of unknown cause.

Then we had our greatest good fortune. With more experience of the use of fine X-rays and better techniques we began to get better pictures and quite suddenly we were able to distinguish structure within the carious lesion by this means. The results fitted very closely with our findings in polarized light, and we could now see not only the evidence of a loss of calcium salts but also the spaces from which they were lost. And more important still, we could see that calcium salts were being lost from some structures within the enamel and not from others. We already knew that this happened at the enamel surface when the small points of entry were opened up and it was necessary to explain these variations in the susceptibility to decalcification of the various structures in the enamel.

There are only two main components of the enamel—the calcium salts and the organic or protein part—and it seemed almost certain that one or the other was responsible for the difference in the susceptibility of the various structures to the removal of calcium salts.

The most probable cause seemed to be a difference in the calcium salts in the various structures but all the evidence suggested that the same calcium salt was present in all structures.

This left the organic or protein material as the other possible factor. It had recently been shown by one of our colleagues (Stack 1954) that this consists of two parts, one a keratin, which is very insoluble, and the other which has as yet no name except "soluble protein". Further investigation showed that the probable distribution of the two substances seemed to correspond closely with the distribution of the two groups of structures which were susceptible or resistant to the removal of calcium salts.

A new piece of information was then published by Rowles (1955). If one takes powdered enamel and treats it with dilute acid one would have expected the calcium salts to be dissolved, but it was shown that the first material to be dissolved was an organic substance which was almost certainly the soluble protein. After this some calcium salts were dissolved fairly quickly. Gradually the remainder of the calcium salts went into solution leaving finally a residue of insoluble keratin.

This was all we needed. We could now explain all our findings. It is too early to be certain, but it seems highly probable that the small crystals of calcium salts are embedded in or coated by the organic material, some by keratin and some by soluble protein. The surface layer probably consists chiefly of crystals coated with keratin, but at certain points a few crystals coated with soluble protein reach to the surface. The attack of caries first dissolves the soluble protein exposing the crystals within to solution, while the keratin protects its crystals for a very much longer time. Thus the stages of caries previously mentioned can now be explained.

First spaces are produced, probably by the removal of soluble protein. This uncovers some of the crystallites of calcium salts and allows them to be dissolved. After this the keratin is attacked and begins to show recognizable histological changes. This process probably also results in the solution of the crystallites from within the keratin. Thus it seems that the initial attack may be not a decalcification but a solution of soluble protein.

Such a theory can explain several of our previous difficulties. It places the structure of the enamel of the individual tooth as a very important factor in deciding whether caries shall occur there or not. It also explains how the surface zone of the enamel can remain largely resistant to attack because its organic matrix is largely keratin and insoluble.

It could also explain the apparent anomaly that badly calcified teeth develop little caries because the important factor in starting the lesion is not the calcium salts but the nature of the protein, and already it can be shown that the protein of the enamel in these teeth shows differences from the normal.

The next question is what causes the attack? There seems no good reason to change our ideas and we still think in terms of a dilute acid produced by the breakdown of refined sugars and carbohydrates by bacteria.

Finally comes the question of treatment and prevention, in the light of our findings.

If our theory is true it may be possible to fill the spaces in the early lesions when they are accessible with some inert material, allowing it to be drawn in by capillary attraction after drying. This might be possible and would probably prevent further progress of the lesions.

But more important still, if the theory is correct we must stop looking for some means of making the calcium salts insoluble. What we require is a means of making the soluble protein insoluble. If this can be done we may well be able to prevent dental decay.

Finally, the real answer would be to learn more of these points of entry which seem to be developmental faults, so that their occurrence might be prevented in development.

Of course so much of this is conjecture that it may all be wrong, but at least we know more of the pathology of caries than we did, and there is some encouragement, for within recent months we have been able to produce artificial lesions, in extracted teeth, which are almost identical with natural decay by using a solvent which attacks only the soluble protein.

### REFERENCES

Bodecker, C. F. (1906) *Dental Review*, **20**, 317.
Miller, W. D. *Micro-organisms of the Human Mouth*, S. S. White Dental Mfg. Co. 1890.
Rowles, S. L. (1955) *J. Dent. Res.*, **34**, 778.
Stack, M. V. (1954) *J. Amer. Dent. Assoc.*, **48**, 297.

## Figures and Tables

**Fig. 1. f1:**
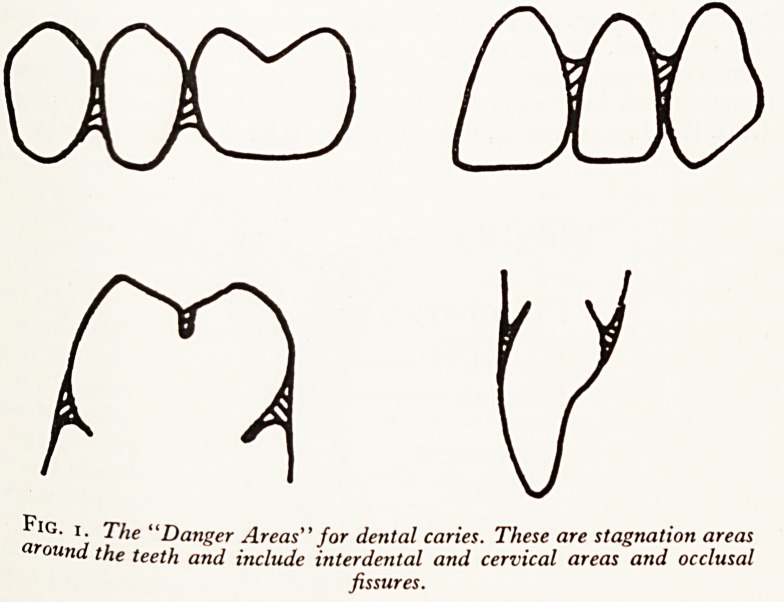


**Fig. 2. f2:**
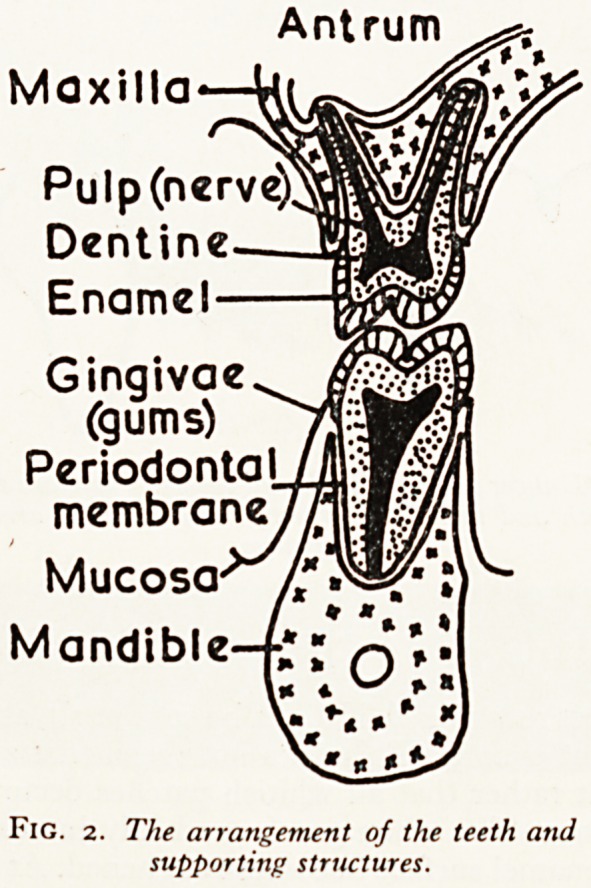


**Figure f3:**
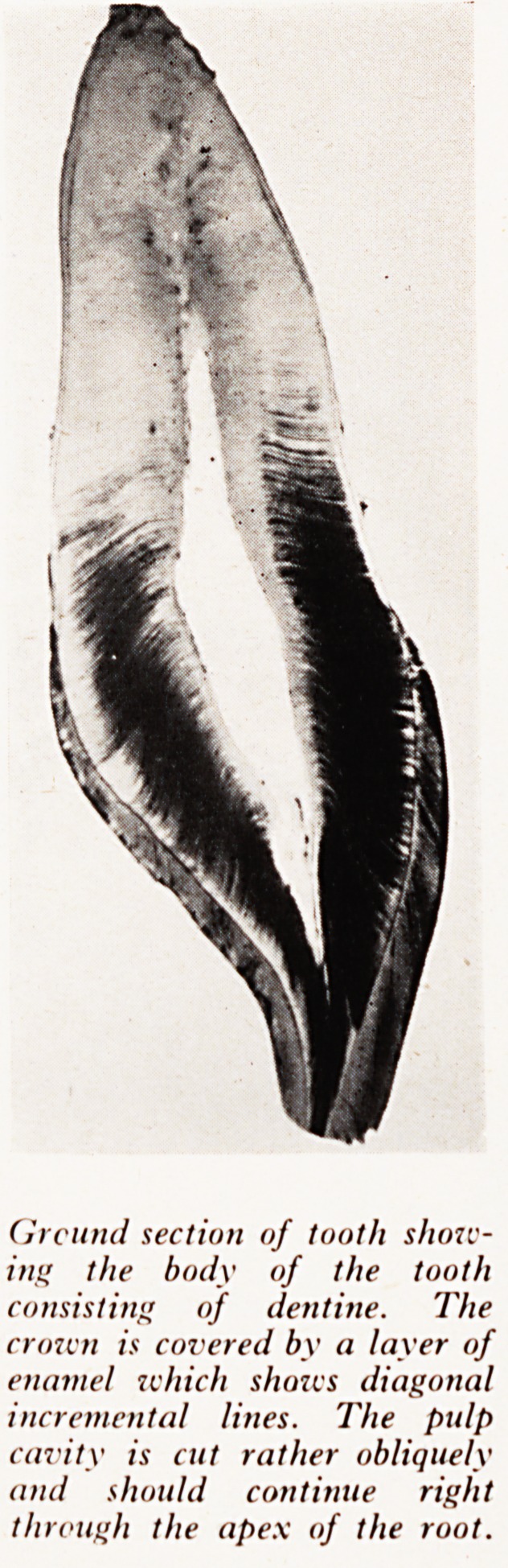


**PLATE XXIII f4:**
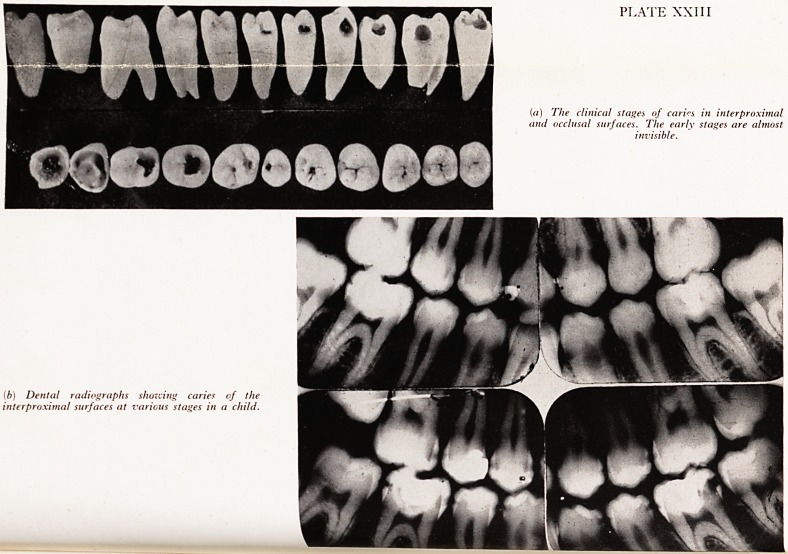


**(a) f5:**
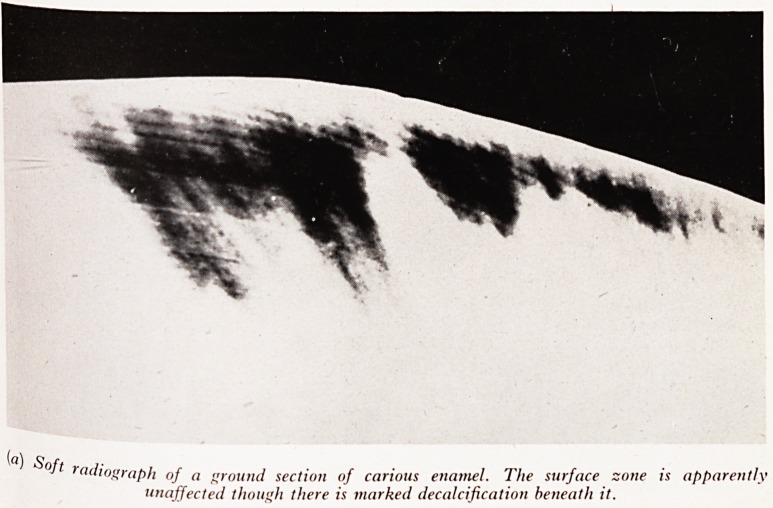


**(b) f6:**
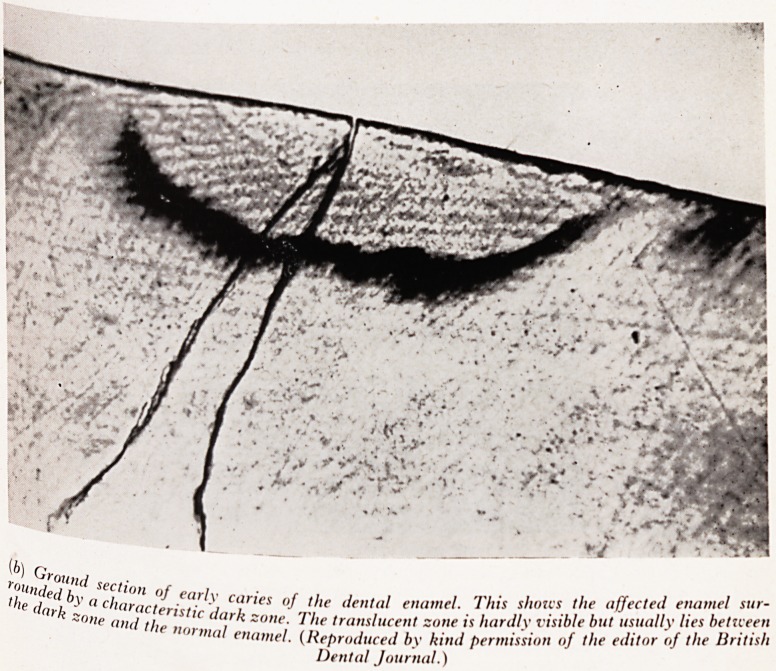


**(a) f7:**
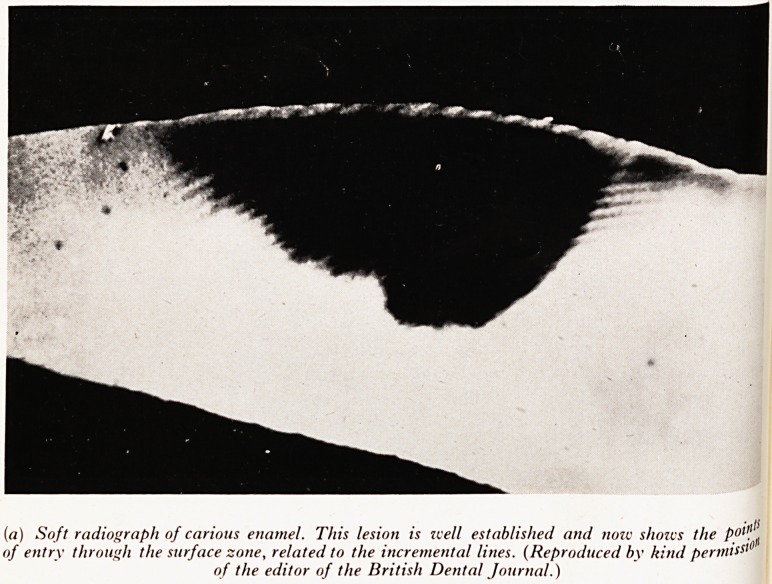


**(b) f8:**